# Artificial Intelligence Will Transform Cardiac Imaging—Opportunities and Challenges

**DOI:** 10.3389/fcvm.2019.00133

**Published:** 2019-09-10

**Authors:** Steffen E. Petersen, Musa Abdulkareem, Tim Leiner

**Affiliations:** ^1^Barts Heart Centre, Barts Health NHS Trust, London, United Kingdom; ^2^NIHR Barts Biomedical Research Centre, William Harvey Research Institute, Queen Mary University of London, London, United Kingdom; ^3^Department of Radiology, University Medical Centre Utrecht, Utrecht University, Utrecht, Netherlands

**Keywords:** artificial intelligence, cardiac magnetic resonance (CMR), deep learning, cardiac imaging, echocardiagraphy, cardiac CT angiogram, cardiac nuclear imaging

## Abstract

Artificial intelligence (AI) using machine learning techniques will change healthcare as we know it. While healthcare AI applications are currently trailing behind popular AI applications, such as personalized web-based advertising, the pace of research and deployment is picking up and about to become disruptive. Overcoming challenges such as patient and public support, transparency over the legal basis for healthcare data use, privacy preservation, technical challenges related to accessing large-scale data from healthcare systems not designed for Big Data analysis, and deployment of AI in routine clinical practice will be crucial. Cardiac imaging and imaging of other body parts is likely to be at the frontier for the development of applications as pattern recognition and machine learning are a significant strength of AI with practical links to image processing. Many opportunities in cardiac imaging exist where AI will impact patients, medical staff, hospitals, commissioners and thus, the entire healthcare system. This perspective article will outline our vision for AI in cardiac imaging with examples of potential applications, challenges and some lessons learnt in recent years.

## Introduction

It is almost impossible in modern society to avoid exposure to artificial intelligence (AI) solutions such as facial recognition, speech recognition, spam filters, chatbots, and personalized web-based advertising. While healthcare AI applications are trailing behind these widely used tools, AI and machine learning in healthcare research is accelerating and about to change healthcare as we know it.

The terms AI, machine learning (ML), and deep learning (DL) are often used interchangeably but are essentially hierarchical. AI is the overarching concept aiming to develop computers with human intelligence. ML methods provides a set of tools to achieve AI and includes supervised, unsupervised and reinforcement learning. DL is a supervised ML technique using neural networks which are used frequently in novel AI solutions.

Through AI clinical pathways will be streamlined and transformed. Opportunities exist at every step, from patient identification and referral to scheduling, image acquisition and reconstruction, automating analysis of images and image interpretation, report writing and communicating the obtained information to the referring physician with clinical decision support systems. There are a number of reasons why AI in healthcare has not been the first area of focus, including data availability, privacy-concerns, and lack of firm and universally agreed upon legal basis for use of data, to name just a few. However, despite these limitations there is substantial untapped potential to transform patient pathways and improve the value of healthcare. Embedding the best AI solutions into clinical practice will likely result from public-private partnerships.

## Success Stories to Date

AI in cardiac imaging is a fast-moving field and is already starting to have an impact clinically. Two factors contributed significantly to the fact that recently a sizeable number of approaches to cardiovascular magnetic resonance (CMR) segmentation algorithms have been published (1–10). Firstly, the National Institute of Health (NIH) initiated the Second Annual Data Science Bowl (2015) on Kaggle, which challenged participants to create an algorithm to measure end-systolic and end-diastolic volumes in cardiac MRIs automatically. Secondly, it was only just over 2 years ago at the 2017 Society for Cardiovascular Magnetic Resonance Annual Scientific Session during which Robert (Bob) Balaban, NIH, challenged the CMR community during his visionary keynote lecture “What's Next To come in CMR” with his slide “Drawing circles. This has to stop.” Of note, companies increasingly offer AI based automated segmentation solutions, with Arterys having received FDA approval for heart MRI interpretation in January 2017 (11).

However, AI applications emerge in all non-invasive cardiac imaging modalities and not just CMR covering a range of applications from image classification, image reconstruction, automation in segmentation and quantification and guiding diagnosis and prognosis. Recently, these have been reviewed comprehensively (12, 13). Cui et al. developed an AI approach to improve image reconstruction for dynamic PET (14). Deep learning solutions to automate calcium scoring from CT attenuation correction in patients following Rb-PET/CT is promising. There was good agreement for the classification into five categories from very low to very high calcium scores (Cohen's kappa up to 0.89) (15). Deep learning approaches have also been shown to diagnose obstructive coronary artery disease more accurately compared to conventional methods using myocardial perfusion imaging (MPI) (16). For SPECT-MPI, a machine learning approach including clinical information was found capable of superior MACE prediction compared to machine learning approaches alone or conventional clinical assessment of SPECT-MPI (16, 17).

A recent study in over 14,000 echocardiograms showed promise for AI applications to automatically classify image orientation, perform image segmentation and assign a diagnosis (18). Detection of wall motion abnormalities on echocardiography requires experience and can thus suffer from observer variability. AI solutions are emerging which have good accuracy for this task (19). Furthermore, in the Multi-Ethnic Study of Atherosclerosis (MESA), Ambale-Venkatesh et al. demonstrated the potential of machine learning to develop more accurate cardiovascular risk prediction tools when supplied with clinical information and data from deep cardiac imaging phenotyping, such as CMR, cardiac CT and carotid ultrasound (20).

## Clinical Opportunities With AI in Cardiac Imaging

Despite these success stories of use of AI in cardiac imaging, we are only at the beginning of a journey. Imaging (including cardiac) is likely to be at the frontier for the development of AI applications as pattern recognition is a significant strength of AI. AI solutions in cardiac imaging will impact the entire healthcare system. This will happen through the range of beneficiaries including patients, medical staff, hospitals, and commissioners. One way to structure these opportunities is to consider the patient pathway involving non-invasive cardiac imaging. It does not take much imagination to identify tasks that AI may tackle efficiently along the entire pathway from patient identification and referral to informing patient management based on the cardiac imaging report. We believe AI will tackle well-circumscribed tasks initially, but the ultimate aim should be to integrate the separate tasks into one smooth and efficient pipeline. This integration of tasks is an aim of the CMR programme “SmartHeart: Next-generation cardiovascular healthcare via integrated image acquisition, reconstruction, analysis and learning” funded by the Engineering and Physical Sciences Research Council (21).

**Figure 1:**
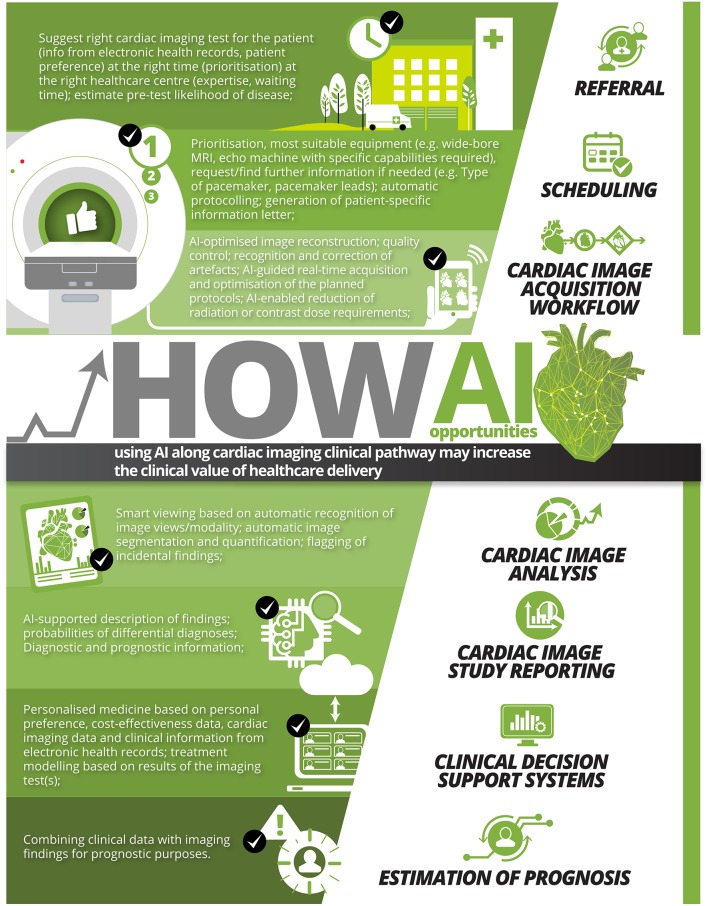
Examples of AI opportunities using AI along cardiac imaging clinical pathway that may increase the clinical value of healthcare delivery.

We list examples of AI opportunities along the clinical pathway in non-invasive imaging in Figure 1.

## Research Opportunities With AI in Cardiac Imaging

Development of clinically robust AI algorithms demands large, high-quality datasets. Slomka et al. set up a large-scale powerful resource for AI development which may serve as template for other non-invasive imaging modalities: REFINE SPECT—the Registry of Fast Myocardial Perfusion Imaging with NExt generation SPECT (22). REFINE SPECT is a multi-center registry with sites contributing nuclear images alongside clinical data in a framework that ensures appropriate de-identification (pseudo-anonymisation) with standardized analysis of all images and follow up for MACE. This collaborative registry will aid development and validation of AI tools for automated diagnosis and risk stratification.

Meta-analyses combine data from multiple studies aiming to summarize and identify common effects or find explanations for variation in the effects observed. Individual participant data meta-analysis compared to study level meta-analysis has many advantages, as discussed in detail by Riley et al. (23). For cardiac imaging, the attraction is that heterogeneity through different approaches to image analysis can be removed. Time-consuming image analysis can now be tackled using AI solutions and opens an opportunity to consider such individual participant meta-analyses. One such application could be the generation of new reference values for common clinical measures, such as LV and RV volumes and mass, with larger datasets. This may allow more precision but also the ability to create much more detailed references ranges, e.g., between ethnicities, age or sex. Another application maybe randomized controlled trials that use cardiac imaging as endpoints which could also be re-analyzed to summarize findings across studies.

Radiomics provide a novel approach to image interpretation, enabling derivation of a multitude of quantifiable metrics related to e.g., signal intensity, texture, shape. Radiomics approaches have been promising in cancer diagnosis and risk stratification. Cardiac radiomics are in their infancy with significant potential impact. Some of these measures are beyond what can be appreciated visually by human experts. In cardiac imaging radiomics may help get closer to personalized medicine through improved diagnosis and risk stratification. A review for cardiac CT radiomics provides an outline of the methodology and opportunities but is applicable to all cardiac imaging modalities (24).

Research is also required to demonstrate the real-world benefit in terms of clinical effectiveness using e.g., quasi-experimental study designs. Furthermore, economic evaluations are required to demonstrate the cost-effectiveness of AI solutions compared to standard of care.

## Challenges

Overcoming challenges such as patient and public support, transparency over the legal basis for healthcare data use, privacy preservation, technical challenges related to accessing large-scale, expertly labeled data from healthcare systems not designed for Big Data analysis, and deployment of AI in routine clinical practice will be critical. These challenges are substantial and only if addressed pro-actively will not lead to substantial slowing down of the AI developments in cardiac imaging.

Future Advocacy, an AI think tank in the UK, has identified challenges of AI and healthcare in a Wellcome Trust commissioned white paper (25). They highlighted that we cannot simply assume patients and the public are happy for healthcare data to be shared and used for AI development. Future Advocacy tasked YouGov to explore patient and public perception. YouGov in 2017 used a representative online survey amongst 2,108 UK adults. Responses were surprising to the question “How comfortable would you be with your personal medical information being used to improve healthcare?”: 49% were uncomfortable with the idea and 11% did not know (25). These findings underscore the need to educate all stakeholders on the potential benefits and risks of using AI techniques. Nundy et al. reflected on how trust between physicians and patients can be enhanced in this era of digital innovation and artificial intelligence. AI in healthcare has the potential to both positively and negatively influence the three components key to trust: competence, motive and transparency. One practical recommendation when implementing AI tools according to Nundy et al. is to strive toward acknowledging the source of the data, qualify AI-based recommendations, and be explainable (26).

Examples of AI have emerged demonstrating the risk of introducing biases into the AI algorithms—from discrimination (27) to adding or removing evidence of medical conditions from medical scans (28). One could imagine if cardiac imaging AI algorithms get trained only on the wealthy population's images that algorithms may then not generalize well to the poor population. This introduces inequalities in health and healthcare. The opposite may also be true—through standardization and availability of AI tools supporting healthcare in poorer parts of the world health, inequalities may be reduced.

Lack of interpretability (“black box”) of the AI solution is not such a concern for the segmentation problem in cardiac imaging. The physician does not need to understand how the segmentation was derived by the AI algorithm as she/he can and should check for accuracy and if needed modify the contours. The responsibility firmly remains with the physician. However, AI tools that predict risk or suggest differential diagnoses may be problematic due to lack of explainability. Removing black boxes in AI is an area of active research to reduce this uncertainty of how decisions are reached—this was the topic of an entire workshop at the Medical Image Computing and Computer Assisted Intervention conference (MICCAI) 2018. Another important question is where the legal responsibility lies when the AI tool makes harmful recommendations. Is it the company providing the AI tool, the physician seeing the patient, the researchers who labeled the training and testing datasets?

There are further challenges and questions related to AI in healthcare and cardiac imaging. In the regulation of AI solutions, the evidence required differs between regulators. How should regulators handle the need to update the AI algorithms over time. How should AI algorithms be checked for reproducibility? How can AI algorithms for the same problem be best compared to identify the optimal solution? Who should be certifying the suitability of AI algorithms for imaging? The Data Science Institute of the American College of Radiology offers the “Certify-AI” service for this purpose to benchmark AI solutions before going to the regulators (29, 30).

There is also little guidance currently on how much data is enough data for developing specific AI tools. As annotation of imaging is time-consuming and expensive, the aim should be to use the minimum data to develop and test robust AI tools. Should the data for training be heterogenous or representative? What is the best trade-off between accuracy and generalizability of the AI tools?

One of the concerns of the public may be related to the sharing of healthcare data with commercial partners for the development of AI tools. Should sharing with commercial partners require prospective consent from patients or opt-out solutions? A key will be to work with patients and the public to build and maintain trust to find practical and acceptable solutions.

If patients withdraw consent for the use of their data in the development of AI algorithms but also from the input it had in developed AI tools, this is a challenge. It is hard to remove single patient datasets from the trained algorithm once it is there, so there is some “non-reversibility” challenge. Some ethicists have argued that since patients do not pay the true price of healthcare, it is not immoral to suggest there is some burden on them to contribute data if this can improve the efficiency of the healthcare system (this operates from the principle that healthcare data are a public good and are neither 100% the property of the patient nor the researcher/institute) (31).

## Lessons Learnt and Key Messages

- Multidisciplinarity is key to success and in an iterative fashion—handing over data/annotated data to the data scientist is not sufficient as a multi-disciplinary approach.
- We need to train imagers to become data experts/AI enablers and AI experts to understand the clinical needs/challenges/risks.
- We need to engage to find solutions of getting the balance right for data access guarding issues related to privacy, ethics, and commercial partnerships.
- Importance of perception. As a community we need to take public and patients and our profession with us on this journey.
- AI is not the solution to everything in cardiac imaging—thus we still need imagers in years to come.

## Vision for AI and Cardiac Imaging

Cardiac imaging will benefit from integration of various AI solutions. Physicians will then be able to focus on tasks best done by human intelligence without being distracted by tasks that can be automated. This may lead to higher physician job satisfaction, improved patient-physician relationships, improved quality of healthcare delivery and improved equality of health and access to healthcare whilst limiting the unsustainable rise in healthcare expenditure in an aging population.

## Conclusion

This era of AI in healthcare is providing disruptive opportunities to transform how we provide non-invasive cardiac imaging in clinical pathways. However, many challenges remain and need to be proactively addressed to keep patients and the public open to the development and introduction of AI solutions. The potential is exciting to make our healthcare systems better in terms of quality, equality and cost-effectiveness.

## Author Contributions

SP, TL, and MA agreed on the outline of the mini-review article. SP drafted the first version. MA and TL contributed to the content and writing of the final version of the manuscript.

### Conflict of Interest Statement

The authors declare that the research was conducted in the absence of any commercial or financial relationships that could be construed as a potential conflict of interest.
